# Neighbourhood natural space and the narrowing of socioeconomic inequality in children's social, emotional, and behavioural wellbeing

**DOI:** 10.1016/j.wss.2021.100051

**Published:** 2021

**Authors:** Paul McCrorie, Jonathan R Olsen, Fiona M Caryl, Natalie Nicholls, Rich Mitchell

**Affiliations:** MRC/CSO Social and Public Health Sciences Unit, University of Glasgow, Berkeley Square, 99 Berkeley Street, Glasgow G3 7HR, Scotland

**Keywords:** Greenspace, Natural Environment, Children, Inequality, Wellbeing

## Abstract

Introduction: The natural environment may benefit children's social, emotional and behavioural wellbeing, whilst offering a lever to narrow socioeconomic health inequalities. We investigated whether immediate neighbourhood natural space and private gardens were related to children's wellbeing outcomes and whether these relationships were moderated by household income.

Methods: A nationally representative sample of 774 children (55% female, 10/11 years old) from the Studying Physical Activity in Children's Environments across Scotland study. Social, emotional and behavioural difficulty scores (Strengths and Difficulties Questionnaire) represented wellbeing outcomes. Percentage of total natural space and private gardens within 100m of the child's residence was quantified using Ordnance Survey's MasterMap Topography Layer®. Linear regression, including interaction terms, explored the two main research questions.

Results: A 10% increase in residential natural space was associated with a 0.08 reduction (-0.15, -0.01; 95%CI) in Emotional Problem scores and a 0.09 improvement (0.02, 0.16; 95%CI) in Prosocial Behaviour scores. Household income moderated the associations between % natural space and private gardens on Prosocial Behaviour scores: for natural space, there was a positive relationship for those in the lowest income quintile (0.25 (0.09, 0.41; 95%CI)) and a null relationship for those in the highest quintile (-0.07 (-0.16, 0.02; 95%CI)). For private garden space, there was a positive relationship for those in the highest quintile (0.15 (0.05, 0.26; 95%CI)) and negative relationship with those in the lowest quintile (-0.30 (-0.50, -0.07, 95%CI)).

Conclusion: The natural environment could be a lever to benefit those from less advantaged backgrounds, particularly the development of prosocial behaviours.

## Highlights

• Natural space (NS) within 100 m of home is negatively associated with emotional problems
• NS within 100 m of home is positively associated with prosocial behaviours
• Relationship between NS and prosocial behaviours stronger in low-income families
• Whereas private garden space more beneficial in children from high-income families
• Increasing residential NS may help narrow inequalities in health and wellbeing

## Introduction

Nature can be defined as the “physical features and processes of nonhuman origin… the ‘living nature’ of flora and fauna” (Hartig et al., 2014, p.208). Contact with nature may provide an opportunity to regulate emotions (Hayball et al., 2018), reduce stress (Ulrich, 1983), and mentally and psychologically recover one's capability to focus attention (Kaplan & Kaplan, 1989).

Multiple literature reviews have evaluated the impacts and associations of the natural environment on children and young people's health and wellbeing (Norwood et al., 2019; Mygind et al., 2019; Tillmann et al., 2018; Twohig-Bennett & Jones, 2018). Previous qualitative work as early as 1997 demonstrated how depriving children of contact with nature can result in frustration, helplessness, and withdrawal, leading to wild, uncontrollable, and dangerous externalised emotional outbursts and tantrums (Bartlett, 1997). Children who live near, or spend time in, natural space have fewer reported social, emotional, and behavioural problems (Tillmann et al., 2018). Nature close to home has also been associated with lower emotional symptom scores in children aged 7-10 years (Amoly et al., 2014), and improved prosocial behaviours in young children aged 4-6 years old (Richardson et al., 2017). Moreover, Markevych et al. (2014) measured access to greenspace (distance to) and behavioural problems in 1,932 children and found a positive association between distance and hyperactivity/inattention. The recent review by Norwood and colleagues (2019) supports the position that homes with higher surrounding greenness may be capable of promoting improved attention in young people.

From a mechanistic perspective, multiple complimentary theories relating to the current paper have been proposed. For instance, Attention Restoration Theory (Kaplan and Kaplan, 1989) has been used to explore the management of symptoms associated with ADHD. This theory proposes a mechanism whereby natural elements in the environment restore the ability to focus directed attention by giving certain neuro-cognitive resources the ability to rest and recover (nature draws one's attention effortlessly). In relation to ADHD, parents involved in previous research have reported less severe symptoms in their children when playing in greener play areas, and improved functioning when engaging in activities in greener settings (Faber Taylor and Kuo, 2011). According to Wells and Evans (2003), attentional restoration inhibits the urge to respond to potentially distracting stimuli, enables attentional focus, and provides the basis through which children can develop resilience and manage life's stresses more effectively. In this sense, contact with nature can act as a buffer, moderating the impact of adverse conditions (e.g. chronic noise, pollution, poor housing quality) and protecting children's psychological, cognitive, and physiological wellbeing.

Similarly, the social affordances provided by nature, whereby the natural environment offers opportunities for social interaction, connectedness, and social support may moderate the impact of stress on children's emotional and behavioural outcomes. Faber Taylor and colleagues (1998) for instance, found that the green spaces were more supportive of children's play and that children had more access to adults in greener outdoor spaces than in the relatively barren spaces. Areas with natural landscaping appeared to promote opportunities for social interaction and support neighbourhood social ties.

The natural cognitive and behavioural development that takes place as children age makes it important to recognise the potential differential role of nature at different age groups. For example, children are likely to spend more time outdoors unaccompanied by an adult, and with other peers, at age 10 than age 4. Additionally, more complex cognitive abilities emerge as children age such as exerting self-control (Diamond, 2006), sharing and social decision-making (Flook, Zahn-Waxler, & Davidson, 2019), and inhibiting selfish impulses (Fehr & Fischbacher, 2003).

Despite a large quantity of primary studies, and numerous reviews, we have identified a notable lack of evidence addressing the potential role of the natural environment in reducing socioeconomic inequalities in health and wellbeing of children and young people – particularly social and emotional factors.

Although complex, the natural environment can be considered an ‘upstream’ determinant and has the potential to improve population level health and contribute to the reduction in socioeconomic-related inequalities (Lucyk & McLaren, 2017). “Equigenesis” describes the disruption of the usual process through which socioeconomic position is converted to health status, thereby reducing or constraining socio-economic health inequalities (Mitchell, 2013). An equigenic environment can theoretically ‘level up’ or ‘level down’: by levelling up, the environment may support the wellbeing of those less advantaged as much as, or more than, the more advantaged; by levelling down, the environment may limit the health of the more advantaged to a greater extent than the less advantaged.

Previous research in adults has demonstrated that differences in mortality incidence rates between the least and most income deprived groups are lowest in the greenest areas (Mitchell & Popham, 2008), and socioeconomic inequality in mental wellbeing is narrower among those reporting better access to recreational/green areas (Mitchell et al., 2015). Although some studies exist on very early lifecourse outcomes (e.g. in utero development and subsequent birthweight and head circumference: Agay-Shay et al., 2014; Dadvand et al., 2015) and younger children (3–7 year olds: Flouri, Midouha and Joshi, 2014; Richardson et al., 2017), none have explored the potential moderating influence of socioeconomic characteristics on the relationship between contact with nature and social, emotional, and behavioural adjustment outcomes in older children (e.g. 10–11 year olds).

Employing the parent-reported Strengths and Difficulties Questionnaire (SDQ), the primary aims of this paper were:

• To explore the impact of the immediate neighbourhood natural space on the social, emotional, and behavioural wellbeing of older children.
• To explore the potential moderating role of socioeconomic circumstances on the relationship between neighbourhood natural space and social, emotional, and behavioural wellbeing of older children – i.e. potential ‘equigenic’ relationship.

## Materials and methods

### Population

We drew on the SPACES (Studying Physical Activity in Children's Environments across Scotland) study, a nationwide exploration of place, space, and mobility. The SPACES dataset provided a unique combination of device-measured physical activity levels, geocoded home address locations from which to derive individualised surrounding natural space, and linkage to a wider set of individual and household level data. Briefly, SPACES sub-sampled participants from Growing up in Scotland (GUS); an on-going Scottish cohort study that began in 2004. The original GUS sample (*n*=5217) was derived from the UK Child Benefits records and sampled children to ensure national representativeness across socioeconomic conditions. From a possible 2,402 children who had participated in the 2014/2015 GUS interviews (aged 10/11 years old), 90% (*n*=2,162) of parents consented to be contacted about SPACES. Parents and potential participant children were then sent study information, registration documents, consent forms, and study devices (e.g. accelerometers and questionnaires) by post (McCrorie, Walker and Ellaway, 2018). The data collection for SPACES took place between May 2015 and May 2016 and ethical approval was provided by the College of Social Sciences, University of Glasgow (CSS ref: 400140067).

### Measures

#### Outcome variable – Strengths and difficulties

Emotional and behavioural wellbeing were assessed with the well-validated (Deighton et al., 2014; Mark and Pike, 2017) *parent-reported* Strengths and Difficulties Questionnaire (Goodman, 1997). The questionnaire contains 25 items and comprises five scales of five items each: *Hyperactivity/Inattention, Emotional problems, Conduct problems, Peer Relationship problems*, and *Prosocial Behaviour*. Scores for each scale may range 0-10 if all items were completed. Scores for each scale were calculated if at least three out of five items were completed and scaled up (n=89 in current analysis) pro-rata if less than 5 (e.g. a score of ‘4’ based on three completed items was scaled up to a score of 7 (6.67 rounded up) for 5 items). A *total difficulties* score was generated by summing scores from all scales excluding the prosocial scale and ranged from 0-40. This was counted as missing if one of the four component scores were missing. SDQ data were linked to the SPACES sample from the 2014/2015 GUS data collection.

Internal consistency of the items (Cronbach, 1951) within each SDQ scale were good for Total Difficulties (Cronbach alpha coefficient = 0.81) and Hyperactivity Problems (0.78), acceptable for Emotional Problems (0.68), Peer Problems (0.66), and Prosocial Behaviour (0.61), and poor for Conduct Problems (0.48).

#### Exposure measure - Natural and private garden land cover

**Fig. 1:**
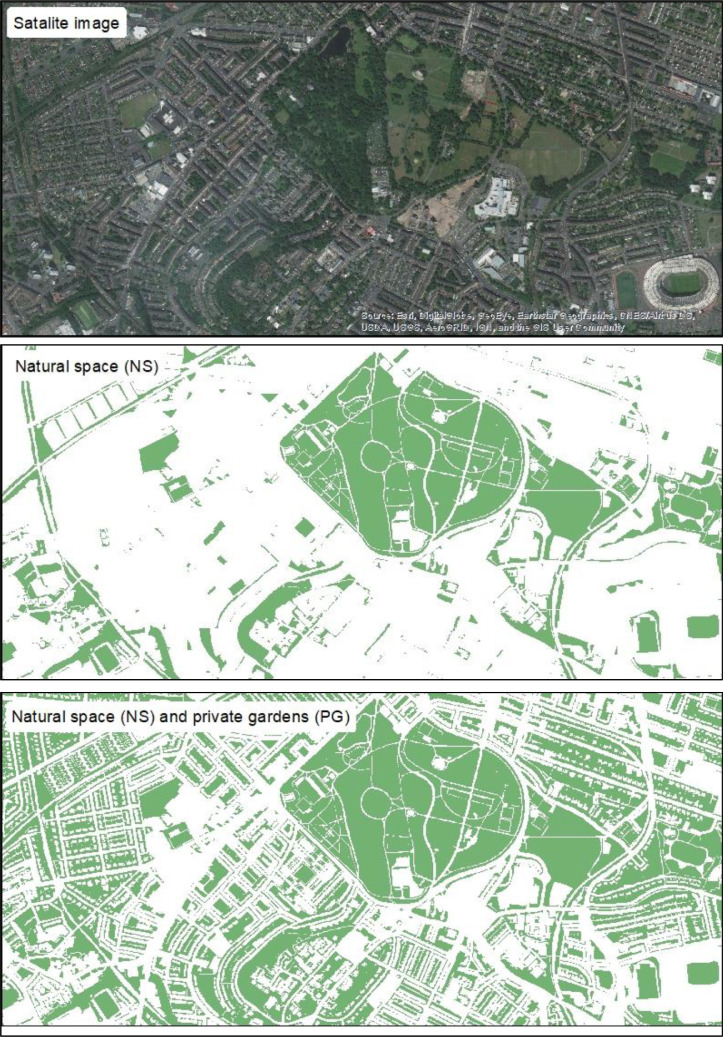
Satellite imagery and digitised representation of the ‘natural space’ (NS) and ‘natural space (NS) and private garden (PG) combined’ layers created and used as our exposure measure.

Digitised land cover data were obtained from the Ordnance Survey (OS) MasterMap Topography Layer® (Ordnance Survey, 2019). OS MasterMap is the most detailed, accurate, and comprehensive geographical data of the UK's landscape. National grid tiles representing the whole of Scotland were imported into ArcGIS 10.3 and two layers created (Fig. 1):

• A layer extracting all land covers classified as ‘Natural’. We extracted features based on the ‘Make’ attribute of feature classifications. This indicates if the feature is man-made or natural (e.g. cliffs, areas of water, (un)cultivated vegetation, trees, marsh, and shrub). We used this attribute classification to be as objective as possible by using the OS operational definition, which will also improve future comparisons. This layer included all accessible formal/non-formal areas within cities and rural areas. A full list of landcovers considered to be natural can be found here (https://www.ordnancesurvey.co.uk/documents/os-mastermap-real-world-object-catalogue.pdf).
• A layer that included natural space (NS) and private gardens (PG; Feature code: 10053). Private gardens are private residential land that combine with - but classed as separate landcover from - the main dwelling to form the overall geographical footprint of a residence. Private gardens are identified as ‘multiple’ Real World Objects (RWOs) within OS MasterMap Topography Layer and can be classified as ‘natural’ or ‘man-made’.

Creating these two layers allowed us to perform analyses separately for i) NS only, ii) PG only, and iii) NS & PG combined. We chose to use the MasterMap Topography layer rather than other sources, such as the Scottish Greenspace Map (Ordnance Survey, 2017), because we needed data from *all* areas in Scotland, not just the urban zones typically covered by those sources.

Recent data from Canadian children (*n*=87, 9-11 years old) supports the immediate neighbourhood as an important behavioural context: on average, 96.5% of children's time was spent close to home where 15% of participants did not venture more than 200 m from their residence (Loebach & Gilliand, 2016). Data from the current study sample also provides support for exploring the immediate environment; approximately 46% of children's time was spent within 50 m of home (Olsen et al., 2019).

As such, a 100-metre circular buffer was calculated for each individual child to represent the most proximal space for social and physical engagement (e.g. physical activity) with nature. It also reflects the potential ‘visual’ mechanistic pathways, specifically the positive mental wellbeing benefits (such as reductions in stress and depressive symptoms of being able to *view* nature from one's own home (Grinde & Patil, 2009; Kaplan, 2001)).

#### Family socioeconomic position

We linked household income data from the 2014/2015 GUS survey to SPACES participants. Equivalised household income was derived using the OECD (Organisation for Economic Co-operation and Development) modified equivalence scale and adjusts household income to reflect the different resource needs of single adults, any additional adults in the household, and children in various age groups (Knudsen, Palmer, & Bradshaw, 2015). Income data was originally collected in a series of bands (e.g. £10,400 to £15,599, £15,600 to £20,799….>£56,000 or more per year) rather than as a scale of specific individual values (e.g. £12,457). Following equivalisation, household income was rendered into quintiles to reflect the censoring of higher incomes.

#### Covariates – additional variables and confounders

Children's levels of PA were included as a potential covariate. Higher levels of PA have been shown to have a positive impact on mental wellbeing (Biddle & Asare, 2011). SPACES participants were asked to wear the validated (Robusto & Trost, 2012; Romanzini et al., 2014) ActiGraph GT3X+ accelerometer over 8 consecutive days during waking hours to measure **total daily physical activity** (PA)**.** The primary ‘overall’ PA measure was the participant's average counts per minute (cpm) per day - a standardised measure of total PA that integrates all movement recorded (including time spent sedentary, light, moderate, and vigorous) as a function of total wearing time of the device (total counts divided by total wear time).

*Other child level factors* included **sex** and **most recent SDQ score** (i.e. past behaviour/score)**.** Both have been shown to predict SDQ outcomes, and in the case of past behaviour/SDQ score this may confound the relationship between natural space and SDQ scores. Other *household level factors* linked included **maternal age** as a known covariate of child behavioural and cognitive outcomes (Tearne et al., 2015). Maternal age was also socially patterned within the current sample (mean age bottom quintile = 26.3 years, SD = 1.2 years vs. top quintile = 32.8 years, SD = 0.4) and so was included as a potential confounder within the income x SDQ interactions. Living in **urban or rural** parts of the country may confound the relationship between availability of natural space and SDQ outcomes through higher levels of greenspace in rural areas. As such, we included a binary indicator or urban/rural dwelling using the Scottish Government's 2-category classification system (Scottish Government, 2018). This defines urbanicity or rurality by the population size of a settlement (greater or less than 3,000 people). Finally, we derived an *environmental factor,* child's ‘**distance from home to school**’ (km), to control for the potential influence of travel (active and passive modes) to school on both exposure to nature, the social environment, and potential influence on SDQ dimensions. With school travel forming an important component of children's daily behaviour, it offers the opportunity to engage with nature, and with family/friends. Active travel behaviours have been associated with psycho-social factors such as social norms, social modelling, and social support in older adolescents (Verhoeven et al., 2016). Moreover, in development of the Model of Children's Active travel (M-CAT; Pont et al., 2011), the authors recognised that events during the school trip trigger a feedback loop where behaviours and experiences can influence/reinforce children's attitudes, beliefs and values, and included examples such as being afraid of being bullied (which may influence emotions or peer problems subdomains of the SDQ). Children who stay closer to school are more likely to commute through active modes (Macdonald et al., 2019). Distance to school was also socially patterned, with those in the lowest income quintile on average staying closer to school (mean = 1.6 km, SD= 0.3 km vs. 2.5 km, SD = 0.3 km, highest income quintile). The network distance (metres) was calculated from children's home location to their school using the gmapsdistance package (Melo & Zarruk, 2017) within R 3.2.0 in February 2018. The software calculated the shortest distance between these two precise geolocations using the Google Maps™ road and path network for a walked journey.

Confounding due to self-selecting into neighbourhoods that afford health enhancing opportunities (i.e. neighbourhoods with greater availability of natural space or private gardens) is often a concern when interpreting findings from environment/health relationships (Lamb et al., 2020). As part of the current data set, we acquired data on whether participants had ever moved home since 2004 and compared the availability of neighbourhood natural space/private gardens in those who had moved home versus those who had not at the time of our analysis. Confounding due to self-selection could be an issue if those who had moved were also more likely to have greater availability of natural space and/or private gardens. Differences in available natural space were non-significant in those that had moved (*n*=352; 27.5%) vs those that had not (*n*=422; 26.6%, *p*=0.6); differences in private garden space were also non-significant for those that had moved (38.5%) vs those that had not (39.4%, *p*=0.4). As such, a variable controlling for self-selection was not included in our statistical models.

### Analysis

#### Geoprocessing of natural and private garden land cover around home

Children's home locations were geocoded using the full address and postal code provided in the SPACES dataset. For each child we calculated the percentage of i) natural space (NS), ii) private garden space (PG), and iii) natural space and private gardens (NS & PG), within the 100m buffer.

#### Statistical analysis

Analyses were conducted using STATA v.14.2 (STATA Corporation, Texas, USA), and accounted for the clustered and stratified survey sample design of the GUS cohort (Bradshaw et al., 2005). Overall alpha was set at 5%, with corrections applied for multiple testing as required. Sampling weights were applied to allow for non-consent to contact, non-consent and non-compliance of those invited to take part. All analyses used complete case data and pairwise deletion across analyses. No imputation was carried out. In total, 774 children (417 girls, 357 boys; mean age 11.1 years) had complete SDQ outcome data; however, ∼7% of these had some missing covariate data, leaving *n*=724-726 (depending on analysis) in the final sample. There were no significant differences in any outcome measure between those included or excluded from analyses (t-test, *p*>0.1 across all outcomes), and missing covariate data (missing = yes or no) were not related to sex (chi square, *p*=0.90), total physical activity (*t*-test, *p*=0.60), BMI (t-test, *p*=0.77), living in urban or rural areas (chi-square, *p*=0.42), or quintile of area level deprivation (chi square, *p*=0.58). See supplementary Table 1 for more detail of those excluded from analyses.

##### Model progression

Separate linear regression models using Weighted Least Squares (WLS) estimation (used instead of Ordinary Least Squares estimation, such that the contribution of each observation to the residual sum of squares is proportional to its population weight (Heeringa et al., 2010)) were fitted to each SDQ sub-scale score (Hyperactivity score, Emotional problems, Conduct problems, Peer problems, Prosocial behaviours) and ‘Total Difficulties’ score as outcome variables. To reduce the potential impact of collinearity between predictor variables (i.e. the different exposure measures), models were conducted separately for NS, PG, and NS & PG at 100 metres. All models satisfied the required assumptions of WLS linear regression when using svyset data (i.e standard errors are robust and resistant to errors produced by deviations from assumptions).

Models progressed through three initial stages, followed by the introduction of interaction terms:

• Unadjusted models (Model 1): regressed the main natural environment exposure variable with SDQ subscale score/total difficulties as the outcome.
• Base models (Model 2): regressed identified covariates on the SDQ subscale score/total difficulties outcome. Covariate variables and level of measurement: most recent SDQ subscale score/total difficulties score (continuous); household equivalised income (five level factor variable with ‘most deprived’ quintile base category); urban/rural dwelling status (0/1; base category = rural); mothers age at birth (years; continuous); distance to school from home (meters; continuous); sex (0/1, base category = boy); physical activity level (mean cpm; continuous).
• Adjusted main effects models (Model 3): Model 2 + % NS, or % PG, or % NS + PG.
• Interaction models (Model 4): Model 3 + inclusion of interaction term exploring the potential moderating relationship of household equivalised income on NS/PG/NS+PG and SDQ subscales and total difficulties. Adjusted (for survey design and multiple comparisons) Wald tests evaluated significance of interaction terms. The results of the interactions that were significant at the corrected alpha level were summarised and presented using the ‘margins’ family commands in Stata.

## Results

We compared our weighted sample with that of the GUS weighted sample to examine representativeness (the GUS weighted sample is broadly representative of the population). The weighting procedure was largely successful across all variables, with only minor differences compared to the entire GUS Sweep 8 sample (See Supplementary Table 2).

On average, 25% of children's 100m home buffers comprised natural space (Table 1). Availability of natural space was not socially patterned (by equivalised income quintile; Adjusted Wald test, F = 1.22, *p*=0.3). Availability of private garden space within the immediate neighbourhood was socially patterned (Adjusted Wald test, F = 2.89, *p*=0.03) with those from highest income quintile having greater access than those from the lowest income quintile (42.4% vs 34.9%, *p*=0.006 for difference).

**Table 1:** Descriptive characteristics of the sample.

Level	Characteristic	Mean (95%CI)	Weighted Count (%)
Individual	Female		415 (53.6)
	Male		359 (46.4)
			
	Total physical activity (cpm)	622.09 (603.24, 640.94)	
			
	SDQ scores		
	Emotional problems	1.40 (1.23, 1.57)	
	Conduct problems	1.10 (0.97, 1.22)	
	Hyperactivity	2.85 (2.61, 3.10)	
	Peer problems	1.02 (0.84, 1.19)	
	Prosocial behaviour	8.85 (8.71, 8.99)	
	Total SDQ	6.37 (5.89, 6.84)	
Household	Equivalised income		
	Bottom quintile		185 (24.8)
	2^nd^ quintile		173 (23.4)
	3^rd^ quintile		147 (19.9)
	4^th^ quintile		115 (15.5)
	Top quintile		120 (16.2)
			
	Mothers age at birth	29.31 (28.46, 30.16)	
			
	Urbanicity		
	Urban		619 (80.1)
	Rural		154 (19.9)
			
	Distance to school (km)	1.84 (1.60, 2.07)	
			
Neighbourhood	Natural space (% within 100m)	25.10 (23.08, 27.11)	
	Private gardens (% within 100m)	38.36 (36.73, 40.00)	

### Main effect analyses

There were no significant adjusted main effects of PG or NS+PG within 100m of home. As such, all main effect results henceforth relate to the analyses conducted using ‘NS only’.

Table 2 presents unadjusted (Model 1) and adjusted (Model 3) % NS coefficients for each SDQ subscale total difficulty score (see supplementary file, Table 3 for full regression tables).

**Table 2:** Regression coefficients and model outputs exploring relationship between % NS within 100 m from home and SDQ scale scores.

**100m Buffer (NS)**	Emotional Problems	Conduct Problems	Hyperactivity/ inattention	Peer Relationship Problems	Prosocial Behaviour	Total SDQ score
**Model 1 ±**§	-0.04 (-0.11, 0.02)	-0.03 (-0.07, 0.02)	0.06 (-0.06, 0.17)	0.00 (-0.07, 0.08)	0.05 (0.00, 0.11)	0.00 (-0.22, 0.24)
						
**Model 3 (Fully Adjusted)**≠						
						
% natural space§	-0.08* (-0.15, -0.01)	-0.03 (-0.08, 0.03)	0.05 (-0.06, 0.16)	-0.03 (-0.12, 0.05)	0.09** (0.02, 0.16)	-0.06 (-0.22, 0.10)
Population R^2^	0.07	0.12	0.17	0.07	0.11	0.52
Observations	726	726	725	724	726	725

*p<0.05; **p<0.01; ***p<0.001

§ Coefficient scaled to reflect change in outcome for every 10-percentage point increase in natural space

± Unadjusted bivariate association between % NS and SDQ outcome

≠ Adjusted for: most recent SDQ subscale score/total difficulties score (continuous); household equivalised income (5 level factor variable with ‘most deprived’ quintile base category); urban/rural dwelling status (0/1; base category = Rural); mothers age at birth (years; continuous); distance to school from home (meters; continuous); Sex (0/1, base category = Boy); physical activity level (mean cpm; continuous).

NS: Natural space extraction only

There were no significant relationships between % NS and any SDQ scale scores in unadjusted models (Model 1). Following adjustment for covariates, there was a significant beneficial relationship between NS and Emotional Problems scores (a 0.08 point reduction for every 10% increase in NS, *p* = 0.024) and with Prosocial Behaviour scores (a 0.09 increase for every 10% increase in NS, *p* = 0.009). Compared to Model 2, the inclusion of NS (Model 3) predicting Emotional Problem and Prosocial Behaviour scores significantly improved the fit and percentage of variance explained by 1 and 2% respectively.

### Two-way interactions: Exposure x household equivalised income

Household income moderated the effects of % NS (Adjusted Wald test, *F* = 3.71, *p=*0.006) and % PG (Adjusted Wald test, *F* = 4.73, *p*=0.002) on Prosocial behaviour scores. No significant relationship was found across the other greenspace exposure measures, or SDQ outcomes when adjusting for multiple comparisons (Table 3).

**Table 3:** Null Hypothesis test (Adjusted Wald tests) results for two-way interaction effects of green exposure type and household Income on SDQ outcome.

	NS	PG	NS & PG
	100m	100m	100m
Emotional Problems	0.080	0.062	0.450
Conduct Problems	0.875	0.973	0.973
Hyperactivity	0.680	0.867	0.187
Peer relationship	0.854	0.925	0.925
Prosocial behaviour	0.006*	0.002*	0.389
Total SDQ score	0.085	0.820	0.041

P values represent the null hypothesis testing results from the two-way interaction analyses.

*p<0.00833, Adjusted alpha to reflect multiple testing

Adjusted for: most recent SDQ subscale score/total difficulties score (continuous); household equivalised income (5 level factor variable with ‘most deprived’ quintile base category); urban/rural dwelling status (0/1; base category = rural); mothers age at birth (years; continuous); distance to school from home (meters; continuous); sex (0/1, base category = boy); physical activity level (mean cpm; continuous).

#### % Natural Space x Household Income on Prosocial behaviour scores

**Fig. 2:**
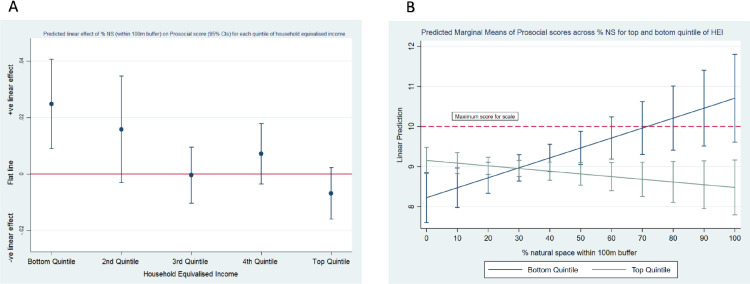
(A) Predicted linear effects of % NS on Prosocial Behaviour Score across levels of Household Equivalised Income (HEI); (B) Predicted margins of Prosocial Behaviour scores for top and bottom quintiles of HEI for every 10% point change in neighbourhood NS (within 100 m)

Fig. 2A demonstrates the predicted linear effects of increasing NS for each income quintile on Prosocial Behaviour scores. The general pattern across income quintiles was one moving from a positive predicted linear relationship (bottom income quintile: linear prediction for a 10% increase in NS = 0.25 (0.09–0.41, 95%CI), *p*=0.003), to a null/slight negative relationship (top income quintile: linear prediction for a 10% increase in NS = -0.07 (-0.16–0.02, 95%CI), *p*=0.144); test for difference between top and bottom linear predictions = 0.32 (0.13–0.50, 95%CI), *p*=0.001. Predicted linear effects were converted into predicted Prosocial Behaviour scores for every 10% increase in neighbourhood NS and presented for the top and bottom quintile (Fig. 2B) and each income quintile separately (supplementary Fig. 1). In a hypothetical situation of zero % natural space within 100 m of home, children from the most affluent income quintiles were predicted to have the highest levels of Prosocial Behaviour scores (marginal mean = 9.15; 8.83-9.48, 95%CI), with those from the least affluent quintile the lowest levels (marginal mean = 8.22; 7.60-8.85, 95%CI); test for difference = 0.93, *p*=0.013. As % NS increases within the 100 m buffer, Prosocial scores were predicted to increase for those in the lowest income group but not for those in the highest income group. Fig. 2B shows the cross-over interaction at approximately 30% of NS.

#### % Private Garden Space x household income on Prosocial behaviour scores

**Fig. 3:**
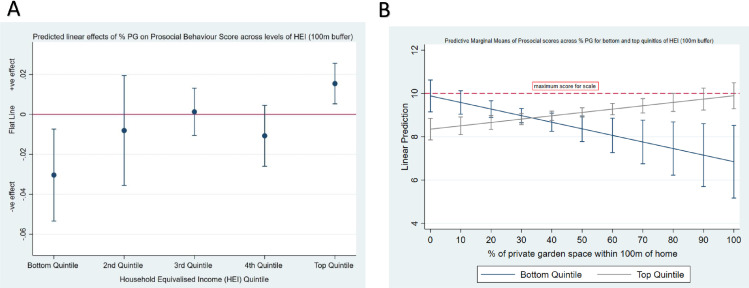
Satellite imagery and digitised representation of the ‘natural space’ (NS) and ‘natural space (NS) and private garden (PG) combined’ layers created and used as our exposure measure

Fig. 3A demonstrates the predicted linear effects of increasing % PG for each income quintile on Prosocial Behaviour scores. The general pattern was one moving from a negative predicted linear relationship (bottom income quintile: linear prediction for a 10% increase in PG = -0.30 (-0.50, -0.07, 95%CI), p=0.01) to a positive relationship (top income quintile: linear prediction for a 10% increase in NS = 0.15 (0.05, 0.26, 95%CI), *p*=0.003); test for difference between top and bottom linear predictions = 0.46 (0.19–0.72, 95%CI), *p*>0.001. As % PG increased within the 100m buffer, prosocial scores were predicted to decrease for those in the lowest income group and increase for those in the highest income group (Fig. 3B). Fig. 3B shows the cross-over interaction at approximately 33% of PG. See supplementary Fig. 2 for each quintile band separately.

## Discussion

Our study examined the potential impact of the immediate neighbourhood natural space on social, emotional and behavioural wellbeing in older children as measured by the Strengths and Difficulties Questionnaire (SDQ). Additionally, we examined the potential ‘equigenic’ role that neighbourhood natural space may have on children's SDQ outcomes. Our results support the position that nearby natural space may have a beneficial association with prosocial behaviour (increase) and emotional outcomes (decrease) in older children. Children spend a considerable amount of time per day in their home neighbourhood; recent work suggests up to 46% of children's time is spent within the immediate home boundary (Olsen et al., 2019). As such, we must consider how the immediate environment encourages children's engagement, including the affordances it offers, and what it means for their social interactions, management of emotions (Hayball, 2018), and other important developmental and wellbeing indicators such as engagement in play (Markevych et al., 2017). On average, children had approximately 25% natural space within their immediate surroundings. A key insight is that the greater benefit of natural space for less advantaged children does not seem to manifest until the proportion of NS within their 100 m buffer moves beyond 30%. If we want to see meaningful benefits for those in greatest need, then the practical implications for policy and planning will transcend both neighbourhood design and new housing development legislation; and perhaps just as importantly, protecting existing useable and accessible natural space from urbanisation.

Mitchell and colleagues (2015) wrote convincingly about identifying macro level characteristics that can disrupt the typical conversion of socioeconomic disadvantage into poorer health outcomes. Prosocial behaviours are inherently altruistic and involve complex cognitive, emotional, and behavioural processes. They are not just moral behaviours, but have genuine impact on mood, reduced risk for depression, self-esteem, and overall mental wellbeing (Alarcon & Forbes, 2017; Schwartz et al., 2003; Snippe et al., 2018; Wilson and Musick, 1999). Where the availability of private gardens seemed to provide greater benefit for those children from more affluent households, and thereby reinforcing positive outcomes for these children, the ‘equigenic’ effect of natural space may prove crucial to preventing, reducing, or at least minimising the widening of health inequality.

The design of our study precludes any detailed evaluation of the mechanisms. Both of the following mechanisms may be equally true: the immediate natural space and neighbourhood private gardens may afford both physical and social properties (i.e. space and place) for the development of prosocial behaviours (e.g. sharing, helping, empathy, and altruism). Likewise, the restorative (Kaplan & Kaplan, 1989) or stress reduction (Ulrich, 1983) properties of nature, where cognitive and physiological properties are given an opportunity to rest and restore, may positively impact children's abilities to manage their emotions. Our results suggest that the pathways through which natural space and private gardens exert their impact may be different for low- and high-income children. Availability of private garden space within the immediate neighbourhood was socially patterned (mean = 42.4% (high income) vs 34.9% (low income)), yet their distributions demonstrated a reasonable range in availability *within* each quintile band (low-income quintile: min = 1.9%; median = 36.6%, IQR = 20.2–47.3%; max = 64.0%; high income quintile: min = 1.2%, median = 43.6%, IQR = 33.2–53.4%, max = 74.6%). The positive effect of private garden space for affluent children (and conversely the negative relationship for poorer children) is therefore not simply one of greater availability. The negative association between private gardens (and positive association with natural space) and prosocial behaviours for those in the lowest income quintile may reflect sociocultural differences in the conception of ‘place’ and engagement with outdoor space. For instance, those in affluent households may use their gardens to engage in play with friends whereas those in lower income households may engage in more community-based play. Previous work by Mass and colleagues (2009) offer support for this pathway in low-income households; greater access to greenspace and reduced risk of poor health may be mediated by the natural environment's ability to encourage social contacts, sense of community, and place identity. Wrapped up in any explanation, however, will also include issues around perceived and objective accessibility, useability, and/or quality of the available space (Mitchell, Africa and Logan, 2018). Furthermore, type of residence (e.g. single storey, multi-storey, detached, semi-detached) and/or residential density could have explained some of the findings, where children staying in high rise apartment blocks may influence the likelihood of using any available private garden space within the immediate neighbourhood. To test this to some degree, we conducted three-way interactions with our urban and rural classification measure (results not shown) to evaluate the direction of the association between private garden space and prosocial behaviours for those in the lowest income quintile. In rural areas of Scotland, housing type is majority single-storey and residential density is comparatively lower than more urbanised areas. Regardless of urban or rural dwelling, the direction of association between private garden space and prosocial behaviours was negative (and conversely positive for those in the highest income quintile), meaning that dwelling type may also not fully explain the impact of private garden space. Future exploration through more refined quantitative measures, in addition to qualitative insight could prove invaluable to understand these relationships further.

When combined into a single exposure measure (NS & PG), the individual (opposing) exposure relationships were nulled; there was no suggestion of a main effect or interaction. Future research should guard against only using combined measures of natural environment (with private gardens) in case opposing effects cancel each other out.

Our findings offer mixed support and consistency with existing literature. In comparison to previous work in England (Flouri et al., 2014), USA (Wells & Evans, 2003), and Spain (Amoly et al., 2014), our findings demonstrated minimal positive impact of residential greenspace on children's wider psychosocial, and behavioural wellbeing. For instance, in an analysis of over 6000 children from the UK, Flouri and colleagues (2014) found that access to a garden and use of parks and playgrounds were related to fewer conduct, peer and hyperactivity problems. Higher neighbourhood greenspace was also related to fewer emotional problems in poorer urban children between ages 3 and 5 years old. Our main effect analyses highlighted similar results for lower emotional problems (full sample) but not for conduct, peer, or hyperactivity problems. Our sample was however slightly older. Importantly for our Scottish context, Richardson and colleagues (2017) found a significant positive effect of natural space on Prosocial Behaviour scores in a larger, urban-only, sample (n=2909) of the same children who participated in the present study when they were between four and six years old. Comparisons across most studies are problematic due to vastly different methodological approaches and study designs, including the measurement of exposure variables (e.g. self-reported naturalness of residential environment; measures of vegetation such as Normalized Difference Vegetation Index), the geographical scale of exposure (e.g. postcode level, Lower Layer Super Output Areas), and the inclusion of urban and rural samples and localities (Labib, Lindley and Huck, 2020).

### Strengths and weaknesses

This was a large study of 774 Scottish children and had a number of strengths. Compared to similar UK work (Richardson et al., 2017) we were able to expand the geographical coverage of the sample and exposure measures. The sample was linked to robust covariates (e.g. household income) in addition to more detailed and specific individual level data collected as part of the SPACES study (such as accelerometry recorded physical activity levels and GIS derived metrics such as distance to school). Our model parameters were unlikely to be biased by the potential for neighbourhood self-selection (Lamb et al., 2020), although we acknowledge that availability of natural or private garden space is not the only factor that could influence choice; *quality* of natural space may be as, if not more, important, of which we were unable to assess or account for.

We obtained a measure of individualised natural space that covered urban and rural areas across a national geographic level from a validated source (Ordnance Survey, 2019). Although our natural environment measure was from 2019, we believe that any natural environmental change from when our data was collected (2016) would have been minimal. OS Mastermap is regularly revised, yet only certain features (primarily manmade features such as buildings and roads) are updated continuously. Changes to the natural environment, which tend to be slower and less evident, are updated periodically from aerial surveying with a revision cycle of between two and ten years (Ordnance Survey, 2021).

Previous research has been limited to area level extraction of greenspace measures (Amoly et al., 2014), which has scaling implications on the exposure-outcome relationship (i.e. the Modifiable Areal Unit Problem). Similarly, there is the known issue of the Uncertain Geographic Context Problem (UGCoP), where researcher-defined areas of exposure (in the operationalisation of the exposure of interest) may not reflect the true environmental experience of every individual (Kwan, 2012). Our environmental context of interest was specific to the immediate environment around the home residence to reflect recent work suggesting that 46% of children's time was spent within 50 m of home (Olsen et al., 2019), to best consider ‘actual’ exposure, and to reflect the younger age of the children involved in this study (i.e where boundaries of indpendent mobility are more restricted; Shaw et al., 2015). We recognise that integrating multiple buffer sizes within the analysis could have been advantageous (Smith et al., 2021), although additional analyses conducted at 800m (results not shown but regression table can be found in supplementary Table 3) revealed no association with any SDQ outcome for either natural space or private garden space.

Moreover, previous research has been limited by the inclusion of *only* urban parks, specific categories of greenspace, or measures of ‘greenness’ using metrics such as the NDVI, causing certain small areas of greenspace to be missed. Using the natural space data allowed us to include children living in urban, semi-rural and remote rural areas, as well as all types of green space, such as green verges and small pockets of natural space that aren't formally recognised as a park. Like many studies, we were unable to examine the *quality* of the natural space at a nationwide level. Although creating a single measure of natural space was a strength of the analyses, it also prevented the deconstruction into its constituent greenspace types (public parks, trees, shrubs, vegetation, uncultivated land etc). Having high-quality, robust information across sub-classifications would allow for more detailed exploration of the interaction effects (e.g. certain sub-types of natural space may be socially patterned in their distribution or certain types of natural space classifications differentially impact children experiencing varying levels of (dis)advantage). A greater insight into these would be of value to policy makers, planners, and practitioners working with children in outdoor settings. Decisions around protecting natural uncultivated areas as opposed to providing new public green spaces for instance have different implications.

Moreover, for this analysis we were unable to identify whether children did or did not *use* the natural spaces within their home environment, and this is a limitation of many studies examining the effect of neighbourhood greenspace and health (Patterson & Farber, 2015).

We explored private gardens in the local area but did not have information about whether the children could access those private gardens. However, it is likely that our private garden measure may also be an indicator of the underlying urban and social fabric of the local area they reside in. For example, a high proportion of private gardens within a 100m buffer of the child's home could indicate a local neighbourhood consisting of multiple dwellings with private gardens. Finally, the study was cross-sectional and as such cannot determine causality. Residual confounding is also possible.

### Future directions

We encourage future research to recognise the potentially different influences of ‘greenspace type’ on wellbeing outcomes, particularly as a function of socioeconomic circumstance. When combined into a single exposure measure (NS & PG), the individual (opposing) exposure relationships were nulled; there was no suggestion of a main effect or interaction. Future research should guard against only using combined measures of natural environment (with private gardens) in case opposing effects cancel each other out.

Embedded qualitative work should be employed to unpack some of the finer meaning behind these different types of green spaces, their physical and social affordances, and their implications for drawing better conclusions on mechanisms of wellbeing impact. ‘Use’ of natural space and private gardens was missing from our analysis and this must be considered in future work testing its importance as a factor on the causal pathway. Previous research has indicated that human/nature relationships can fall under three broad categories: accessibility (e.g. availability in residential area), exposure (e.g. time spent in nature), and engagement (e.g. purposeful interaction that is directed, intentional, and sustained); each potentially offering evidence relating to the dose/response relationship (Tillmann et al., 2018). The present analysis focused primarily within the accessibility field for good reason. We are building a programme of work combining analyses exploring the importance of the home environment, in addition to school and wider environmental exposure that, when taken together, will provide robust and systematically derived evidence to inform future decision making. This paper forms the inception of this journey. Our future work will use detailed mobility data (e.g. GPS data) to examine how both accessibility and use of natural space are associated with mental health outcomes for children. This will also provide an opportunity to explore the importance of accessing greenspace outside of the home neighbourhood, including exposure at, and travel to, school. Finally, like many researchers working in the area, we were unable to include a measure of ‘quality’, and as such, we recommend future research should explore creating a national green or natural space quality indicator.

## Conclusions

A considerable amount of academic thinking and political will has been invested into the identification, exploration, and implementation of strategies to narrow the health and wellbeing disparities resulting from socioeconomic disadvantage. Unfortunately, in a world where our economic systems actively fight to widen social inequalities, this is an uphill battle. Prosocial behaviours are inherently altruistic, and involve complex cognitive, emotional, and behavioural processes. They are not just moral behaviours but have a genuine impact on our mental wellbeing. The findings from this study provide support for using our natural resources as a lever to benefit those in greatest need. Further research should be conducted to explore our findings in detail, including research designs confirming causality, and exploring mechanistic pathways. In doing so, the combined evidence base can better influence decision makers in policy, planning, and implementation.

## **A**uthor contributions

PM secured funding for the SPACES project, project managed the data collection, and conceptualised the paper. PM conducted secondary statistical analysis and wrote the manuscript; JO and FC contributed to the conceptualisation of the paper, conducted all Geographical Information System analyses, and helped write sections of the original draft; NN provided statistical support, conducted all primary formal statistical analyses and contributed sections of writing to the original draft; RM contributed to the conceptualisation of the paper, assisted with the statistical analysis plan, and reviewed and edited the paper. All authors critically revised the article and signed off the final draft.

## Declaration of Competing Interest

The authors declare that they have no known competing financial interests or personal relationships that could have appeared to influence the work reported in this paper.

## Appendix Supplementary materials

Image, application 1

Image, application 2

## Footnotes

Supplementary material associated with this article can be found, in the online version, at doi:10.1016/j.wss.2021.100051.

## References

[bib0001] Agay-Shay K., Peled A., Crespo A.V., Peretz C., Amitai Y., Linn S., Nieuwenhuijsen M.J. (2014). Green spaces and adverse pregnancy outcomes. Occup. Environ. Med..

[bib0002] Alarcon G., Forbes E.E. (2017). Prosocial behavior and depression: a case for developmental gender differences. Curr. Behav. Neurosci. Rep..

[bib0003] Amoly E., Dadvand P., Forns J., López-Vicente M., Basagaña X., Julvez J., Sunyer J. (2014). Green and blue spaces and behavioral development in Barcelona schoolchildren: the BREATHE project. Environ. Health Perspect..

[bib0004] Bartlett S.N. (1997). No place to play: Implications for the interaction of parents and children. J. Child. Poverty.

[bib0005] Biddle S.J., Asare M. (2011). Physical activity and mental health in children and adolescents: a review of reviews. Br. J. Sports Med..

[bib0006] Bradshaw, P., Tipping, S., Marryat, L., & Corbett, J. (2005). Growing Up In Scotland Sweep 1 - 2005 User Guide. Retrieved from Edinburgh: http://doc.ukdataservice.ac.uk/doc/5760/mrdoc/pdf/5760_userguide_cohort1_sweep1.pdf.

[bib0007] Dadvand P., Nieuwenhuijsen M.J., Esnaola M., Forns J., Basagaña X., Alvarez-Pedrerol M., Sunyer J. (2015). Green spaces and cognitive development in primary schoolchildren. Proc. Natl. Acad. Sci..

[bib0008] Deighton J., Croudace T., Fonagy P., Brown J., Patalay P., Wolpert M. (2014). Measuring mental health and wellbeing outcomes for children and adolescents to inform practice and policy: a review of child self-report measures. Child Adolesc. Psychiatry Ment. Health.

[bib0009] Diamond A. (2006). Lifespan cognition: Mechanisms of Change.

[bib0010] Fehr E., Fischbacher U. (2003). The nature of human altruism. Nature.

[bib0011] Flook L., Zahn-Waxler C., Davidson R.J. (2019). Developmental differences in prosocial behavior between preschool and late elementary school. Front. Psychol..

[bib0012] Flouri E., Midouhas E., Joshi H. (2014). The role of urban neighbourhood green space in children's emotional and behavioural resilience. J. Environ. Psychol..

[bib0013] Goodman R. (1997). The strengths and difficulties questionnaire: a research note. J. Child Psychol. Psychiatry.

[bib0014] Grinde B., Patil G.G. (2009). Biophilia: does visual contact with nature impact on health and well-being?. Int. J. Environ. Res. Public Health.

[bib0015] Hartig T., Mitchell R., de Vries S., Frumkin H. (2014). Nature and health. Ann. Rev. Public Health.

[bib0016] Hayball F. (2018).

[bib0017] Hayball F., McCrorie P., Kirk A., Gibson A.M., Ellaway A. (2018). Exploring children's perceptions of their local environment in relation to time spent outside. Child Soc..

[bib0018] Heeringa S.G., West B.T., Berglund P.A. (2010).

[bib0019] Kaplan R. (2001). The nature of the view from home:psychological benefits. Environ. Behav..

[bib0020] Kaplan R., Kaplan S. (1989).

[bib0021] Knudsen, L., Palmer, J., & Bradshaw, P. (2015). Growing Up In Scotland Sweep 8 - 2014-2015 User Guide. Retrieved from Edinburgh: https://growingupinscotland.org.uk/wp-content/uploads/2017/11/GUS-BC1-SW8-User-Guide.pdf.

[bib0022] Kwan M-P. (2012). The uncertain geographic context problem. Ann. Assoc. Am. Geogr..

[bib0023] Labib S.M., Lindley S., Huck J.J. (2020). Spatial dimensions of the influence of urban green-blue spaces on human health: a systematic review. Environ. Res..

[bib0024] Lamb K.E., Thornton L.E., King T.L. (2020). Methods for accounting for neighbourhood self-selection in physical activity and dietary behaviour research: a systematic review. Int. J. Behav. Nutr. Phys. Activ..

[bib0025] Loebach J.E., Gilliland J.A. (2016). Free range kids? Using GPS-derived activity spaces to examine children's neighborhood activity and mobility. Environ. Behav..

[bib0026] Lucyk K., McLaren L. (2017). Taking stock of the social determinants of health: a scoping review. PLoS One.

[bib0027] Macdonald L., McCrorie P., Nicholls N., Olsen J.R. (2019). Active commute to school: does distance from school or walkability of the home neighbourhood matter? A national cross-sectional study of children aged 10-11 years, Scotland, UK. BMJ Open.

[bib0028] Mark K.M., Pike A. (2017). Links between marital quality, the mother–child relationship and child behavior:a multi-level modeling approach. Int. J. Behav. Dev..

[bib0029] Markevych I., Tiesler C.M., Fuertes E., Romanos M., Dadvand P., Nieuwenhuijsen M.J., Heinrich J. (2014). Access to urban green spaces and behavioural problems in children: results from the GINIplus and LISAplus studies. Environ. Int..

[bib0030] Markevych I., Schoierer J., Hartig T., Chudnovsky A., Hystad P., Dzhambov A.M., Fuertes E. (2017). Exploring pathways linking greenspace to health: theoretical and methodological guidance. Environ. Res..

[bib0031] McCrorie P., Walker D., Ellaway A. (2018). The unanticipated challenges associated with implementing an observational study protocol in a large-scale physical activity and global positioning system data collection. JMIR Res Protoc.

[bib0032] Melo, R. A., & Zarruk, D. (2017). Package ‘gmapsdistance’: The Comprehensive R Archive Network (CRAN). Retrieved from https://cran.r-project.org/web/packages/gmapsdistance/gmapsdistance.pdf.

[bib0033] Mitchell, R. (2013). What is equigenesis and how might it help narrow health inequalities? Retrieved from http://cresh.org.uk/2013/11/08/what-is-equigenesis-and-how-might-it-help-narrow-healthinequalities.

[bib0034] Mitchell R., Africa J., Logan A., van den Bosch M., Bird W. (2018). Oxford Textbook of Nature and Public Health: The Role of Nature in Improving the Health of a Population.

[bib0035] Mitchell R., Popham F. (2008). Effect of exposure to natural environment on health inequalities: an observational population study. Lancet.

[bib0036] Mitchell R.J., Richardson E.A., Shortt N.K., Pearce J.R. (2015). Neighborhood environments and socioeconomic inequalities in mental well-being. Am. J. Prev. Med..

[bib0037] Mygind L., Kjeldsted E., Hartmeyer R., Mygind E., Bølling M., Bentsen P. (2019). Mental, physical and social health benefits of immersive nature-experience for children and adolescents: a systematic review and quality assessment of the evidence. Health Place.

[bib0038] Norwood M.F., Lakhani A., Fullagar S., Maujean A., Downes M., Byrne J., Stewart A., Barber B., Kendall E. (2019). A narrative and systematic review of the behavioural, cognitive and emotional effects of passive nature exposure on young people: evidence for prescribing change. Landscape Urban Plan..

[bib0039] Olsen J.R., Mitchell R., McCrorie P., Ellaway A. (2019). Children's mobility and environmental exposures in urban landscapes: a cross-sectional study of 10-11 year old Scottish children. Soc. Sci. Med..

[bib0040] Ordnance Survey. (2017). OS Open Greenspace. Retrieved from: https://www.ordnancesurvey.co.uk/business-government/products/open-map-greenspace.

[bib0041] Ordnance Survey. (2019). OS MasterMap Topography Layer. Retrieved from: https://digimap.edina.ac.uk/webhelp/os/data_files/os_manuals/os-mastermap-topography-layer-user-guide_1_12.pdf.

[bib0042] Ordnance Survey. (2021). OS MasterMap revision policy. Retrieved from: https://www.ordnancesurvey.co.uk/business-government/tools-support/mastermap-topography-support/revision-policy.

[bib0043] Patterson Z., Farber S. (2015). Potential path areas and activity spaces in application: a review. Transp. Rev..

[bib0044] Pont K., Ziviani J., Wadley D., Abbott R. (2011). The model of children's active travel (M-CAT): a conceptual framework for examining factors influencing children's active travel. Aust. Occup. Ther. J..

[bib0045] Richardson E.A., Pearce J., Shortt N.K., Mitchell R. (2017). The role of public and private natural space in children's social, emotional and behavioural development in Scotland: a longitudinal study. Environ. Res..

[bib0046] Robusto K.M., Trost S.G. (2012). Comparison of three generations of ActiGraph activity monitors in children and adolescents. J. Sports Sci..

[bib0047] Romanzini M., Petroski E.L., Ohara D., Dourado A.C., Reichert F.F. (2014). Calibration of ActiGraph GT3X, Actical and RT3 accelerometers in adolescents. Eur J Sport Sci.

[bib0048] Schwartz C., Meisenhelder J.B., Ma Y., Reed G. (2003). Altruistic social interest behaviors are associated with better mental health. Psychosom. Med..

[bib0049] Scottish Government (2018).

[bib0050] Shaw B., Bicket M., Elliott B., Fagan-Watson B., Mocca E., Hillman M. (2015).

[bib0051] Smith M., Cui J., Ikeda E., Mavoa S., Hasanzadeh K., Zhao J., Rinne T.E., Donnellan N., Kyttä M. (2021). Objective measurement of children's physical activity geographies: a systematic search and scoping review. Health Place.

[bib0052] Snippe E., Jeronimus B.F., Aan Het Rot M., Bos E.H., de Jonge P., Wichers M. (2018). The reciprocity of prosocial behavior and positive affect in daily life. J. Pers..

[bib0053] Taylor. A.F., Kuo. F.E (2011). Could exposure to everyday green spaces help treat adhd? Evidence from children's play settings. Appl. Psychol.: Health Well-Being.

[bib0054] Tearne J.E., Robinson M., Jacoby P., Li J., Newnham J., McLean N. (2015). Does late childbearing increase the risk for behavioural problems in children? A longitudinal cohort study. Paediatr. Perinat. Epidemiol..

[bib0055] Tillmann S., Tobin D., Avison W., Gilliland J. (2018). Mental health benefits of interactions with nature in children and teenagers: a systematic review. J. Epidemiol. Community Health.

[bib0056] Twohig-Bennett C., Jones A. (2018). The health benefits of the great outdoors: a systematic review and meta-analysis of greenspace exposure and health outcomes. Environ. Res..

[bib0057] Ulrich R.S., Altman I., Wohlwill J.F. (1983). Behavior and the Natural Environment. Human Behavior and Environment (Advances in Theory and Research) (Vol. 6).

[bib0058] Verhoeven H, Simons D, Van Dyck D, Van Cauwenberg J, Clarys P, De Bourdeaudhuij I (2016). Psychosocial and environmental correlates of walking, cycling, public transport and passive transport to various destinations in flemish older adolescents. PLoS One.

[bib0059] Wells N.M., Evans G.W. (2003). Nearby nature: a buffer of life stress among rural children. Environ. Behav..

[bib0060] Wilson J., Musick M. (1999). The effects of volunteering on the volunteer. Law Contemp. Probl..

