# Supplementary material for: Neighbourhood natural space and the narrowing of socioeconomic inequality in children's social, emotional, and behavioural wellbeing
Source: Wellbeing Space Soc. 2021;2:None. doi: 10.1016/j.wss.2021.100051 (PMC9099293; doi:10.1016/j.wss.2021.100051)
Supplement: Supplementary file 1 [file mmc1.docx]

| Demographic variable | SPACES weighted sample distribution (n=774) | Sweep 8 Growing up in Scotland (GUS) weighted sample distribution (n=3,149) |
| --- | --- | --- |
| Income (per annum)  <3,999 - £9,999  £10,000 - £19,999  £20,000 - £28,999  £29,000 - £37,999  £38,000 - £49,999  >50,000 | 3%  20%  18%  14%  14%  27% | 5%  21%  16%  14%  15%  29% |
| Mothers age at birth (years)  Under 20  20 -29  30 – 39  40 or older | 4%  43%  50%  3% | 7%  41%  49%  3% |
| Marital status  Married  Cohabiting  Single  Widowed  Divorced  Separated | 61%  19%  11%  1%  5%  3% | 68%  15%  9%  1%  3%  4% |
| SIMD quintile (2012)  Most  2^nd^  Middling  4^th^  Least Deprived | 21%  18%  19%  21%  21% | 20%  21%  20%  18%  21% |
| Highest educational qualification in household  No qualification  Lower level Standard Grades or equivalent  Upper level Standard Grades or equivalent  Higher Grades or equivalent  Degree level academic or equivalent  Other | 3%  3%  19%  39%  35%  1% | 6%  4%  19%  33%  38%  0.4% |
| Urban/Rural dwelling  Large urban  Other Urban  Small accessible towns  Small remote towns  Accessible rural  Remote rural | 36%  33%  9%  3%  12%  7% | 38%  32%  10%  3%  13%  4% |
| BMI UK categories  Underweight  Healthy weight  Overweight  Obese | 2%  64%  18%  16% | 2%  64%  15%  19% |

Supplementary File 3: Comparison of weighted sample to known national level SES/demographic distributions
