# Supplementary material for: Neighbourhood natural space and the narrowing of socioeconomic inequality in children's social, emotional, and behavioural wellbeing
Source: Wellbeing Space Soc. 2021;2:None. doi: 10.1016/j.wss.2021.100051 (PMC9099293; doi:10.1016/j.wss.2021.100051)

| \| 100m Buffer \| Emotional Problems \| \| Conduct Problems \| \| Hyperactivity \| \| Peer Relationship Problems \| \| Prosocial behaviour \| \| Total SDQ score \| \| \| --- \| --- \| --- \| --- \| --- \| --- \| --- \| --- \| --- \| --- \| --- \| --- \| --- \| \|  \| NS \| NS & PG \| NS \| NS & PG \| NS \| NS & PG \| NS \| NS & PG \| NS \| NS & PG \| NS \| NS & PG \| \| **Unadjusted±**§ \| -0.04  (-0.11, 0.02) \| -0.12  (-0.25, 0.01) \| -0.03  (-0.07, 0.02) \| -0.06  (-0.15, 0.03) \| 0.06  (-0.06, 0.17) \| 0.00  (-0.18, 0.19) \| 0.00  (-0.07, 0.08) \| -0.04  (-0.19, 0.11) \| 0.05  (0.00, 0.11) \| 0.02  (-0.08, 0.11) \| 0.00  (-0.22, 0.24) \| -0.20  (-0.64, 0.23) \| \| **Adjusted** \|  \|  \|  \|  \|  \|  \|  \|  \|  \|  \|  \|  \| \| Sweep 7 score \| 0.21***  (0.09, 0.33) \| 0.21***  (0.09, 0.34) \| 0.27***  (0.07, 0.25) \| 0.27***  (0.16, 0.38) \| 0.31***  (0.22, 0.41) \| 0.31***  (0.22, 0.41) \| 0.23**  (0.08, 0.38) \| 0.24**  (0.09, 0.38) \| 0.16*  (0.03, 0.29) \| 0.16*  (0.03, 0.29) \| 0.70***  (0.62, 0.78) \| 0.70***  (0.62, 0.78) \| \| Income  (ref: bottom) \|  \|  \|  \|  \|  \|  \|  \|  \|  \|  \|  \|  \| \| 2nd Quintile \| 0.27  (-0.39, 0.93) \| 0.31  (-0.37, 0.99) \| -0.12  (-0.51, 0.28) \| -0.11  (-0.50, 0.27) \| -0.48  (-1.21, 0.26) \| -0.48  (-1.21, 0.26) \| -0.23  (-0.87, 0.41) \| -0.20  (-0.83, 0.44) \| -0.10  (-0.60, 0.39) \| -0.11  (-0.62, 0.40) \| 0.33  (-0.96, 1.62) \| 0.42  (-0.88, 1.72) \| \| 3rd Quintile \| -0.01  (-0.50, 0.49) \| 0.06  (-0.45, 0.57) \| -0.12  (-0.43, 0.20) \| -0.10  (-0.41, 0.21) \| -0.35  (-0.94, 0.25) \| -0.35  (-0.96, 0.25) \| -0.35  (-0.98, 0.29) \| -0.30  (-0.91, 0.32) \| -0.03  (-0.46, 0.41) \| -0.05  (-0.51, 0.41) \| 0.09  (-1.01, 1.19) \| 0.23  (-0.90, 1.35) \| \| 4th Quintile \| -0.04  (-0.51, 0.43) \| 0.02  (-0.45, 0.49) \| -0.35*  (-0.66, -0.03) \| -0.34*  (-0.64, 0.03) \| -0.61∞  (-1.29, 0.07) \| -0.62∞  (-1.31, 0.07) \| -0.58∞  (-1.17, 0.01) \| -0.53∞  (-1.10, 0.04) \| 0.14  (-0.31, 0.59) \| 0.12  (-0.35, 0.59) \| -0.01  (-1.04, 1.01) \| 0.12  (-0.92, 1.17) \| \| Top Quintile \| -0.16  (-0.59, 0.28) \| -0.08  (-0.51, 0.36) \| -0.46**  (-0.77, -0.16) \| -0.44*  (-0.74,-0.15) \| -0.61∞  (-1.23, 0.01) \| -0.62*  (-1.24, 0.01) \| -0.62*  (-1.18,-0.06) \| -0.57*  (-1.11,-0.02) \| 0.22  (-0.25, 0.69) \| 0.19  (-0.30, 0.67) \| -0.36  (-1.43, 0.71) \| -0.21  (-1.29, 0.87) \| \| Urban \|  \|  \|  \|  \|  \|  \|  \|  \|  \|  \|  \|  \| \| (ref:Rural) \| -0.15  (-0.65, 0.35) \| -0.15  (-0.66, 0.36) \| 0.05  (-0.37, 0.26) \| -0.03  (-0.36, 0.29) \| 0.01  (-0.45, 0.48) \| 0.07  (-0.56, 0.42) \| -0.38  (-0.92, 0.15) \| -0.43∞  (-0.93, 0.07) \| 0.35∞  (-0.02, 0.72) \| 0.22  (-0.16, 0.60) \| -0.15  (-0.97, 0.66) \| -0.35  (-1.27, 0.56) \| \| Mothers age at birth of child \| -0.02∞  (-0.04, 0.00) \| -0.02∞  (-0.04, 0.00) \| 0.00  (-0.03, 0.01) \| -0.01  (-0.03, 0.01) \| -0.03  (-0.07, 0.02) \| -0.03  (-0.07, 0.02) \| -0.01  (-0.03, 0.02) \| 0.00  (-0.03, 0.03) \| 0.00  (-0.02, 0.02) \| -0.01  (-0.03, 0.01) \| 0.06*  (0.01, 0.11) \| 0.06*  (0.01, 0.11) \| \|  \|  \|  \|  \|  \|  \|  \|  \|  \|  \|  \|  \|  \| \| Distance to School≠ \| 0.04  (-0.01, 0.09) \| 0.03  (-0.01, 0.08) \| -0.02∞  (-0.04, 0.00) \| -0.02∞  (-0.05, 0.00) \| 0.01  (-0.04, 0.06) \| 0.01  (-0.03, 0.07) \| 0.00  (-0.04, 0.03) \| 0.00  (-0.03, 0.03) \| 0.01  (-0.01, 0.04) \| 0.02∞  (0.00, 0.05) \| 0.04  (-0.03, 0.11) \| 0.05  (-0.02, 0.11) \| \|  \|  \|  \|  \|  \|  \|  \|  \|  \|  \|  \|  \|  \| \| Female (ref: Male) \| 0.16  (-0.23, 0.54) \| 0.13  (-0.25, 0.51) \| -0.13  (-0.37, 0.11) \| -0.13  (-0.38, 0.11) \| -0.81**  (-1.31, -0.31) \| -0.81**  (-1.32, -0.30) \| -0.13  (-0.50, 0.24) \| -0.15  (-0.51, 0.20) \| 0.60***  (0.30, 0.90) \| 0.60***  (0.31, 0.90) \| 0.04  (-0.70, 0.78) \| -0.02  (-0.75, 0.71) \| \|  \|  \|  \|  \|  \|  \|  \|  \|  \|  \|  \|  \|  \| \| Total PA^¥^ \| -0.03  (-0.12, 0.06) \| -0.03  (-0.12, 0.06) \| 0.04  (-0.02, 0.10) \| 0.04  (-0.02, 0.11) \| 0.10*  (0.00, 0.20) \| 0.13*  (0.06, 0.24) \| -0.03  (-0.12, 0.06) \| -0.03  (-0.11, 0.06) \| 0.08  (-0.02, 0.16) \| 0.08∞  (-0.01, 0.17) \| 0.20*  (0.02, 0.37) \| 0.20*  (0.03, 0.37) \| \|  \|  \|  \|  \|  \|  \|  \|  \|  \|  \|  \|  \|  \| \| % natural space^§^ \| -0.08*  (-0.15,-0.01) \| -0.13∞  (-0.28, 0.01) \| -0.03  (-0.08, 0.03) \| -0.03  (-0.14, 0.08) \| 0.05  (-0.06, 0.16) \| 0.02  (-0.18, 0.23) \| -0.03  (-0.12, 0.05) \| -0.09  (-0.23, 0.06) \| 0.09**  (0.02, 0.16) \| 0.06  (-0.05, 0.16) \| -0.06  (-0.22, 0.10) \| -0.24∞  (-0.50, 0.01) \| \| Constant \| 2.06  (0.85, 3.27) \| 2.62  (0.96, 4.28) \| 1.18  (0.46, 1.90) \| 1.27  (0.16, 2.38) \| 2.53  (0.80, 4.27) \| 2.59  (0.33, 4.86) \| 1.98  (0.57, 3.40) \| 2.45  (0.60, 4.30) \| 6.21  (4.55, 7.88) \| 6.21  (4.50, 7.93) \| -1.31  (-3.50, 0.87) \| 0.86  (-2.92, 3.09) \| \| Observations \| 726 \| 726 \| 726 \| 726 \| 725 \| 725 \| 724 \| 724 \| 726 \| 726 \| 725 \| 725 \| \| Population size \| 731 \| 731 \| 731 \| 731 \| 730 \| 730 \| 728 \| 728 \| 731 \| 731 \| 726 \| 726 \| \| F Statistic \| 2.91** \| 2.55* \| 4.66*** \| 4.66* \| 9.69*** \| 9.11*** \| 2.31* \| 2.48* \| 3.31** \| 3.24** \| 28.70*** \| 30.08*** \| \| Population R^2^ \| 0.07 \| 0.07 \| 0.12 \| 0.12 \| 0.17 \| 0.17 \| 0.07 \| 0.08 \| 0.11 \| 0.10 \| 0.52 \| 0.53 \| |
| --- | --- | --- | --- | --- | --- | --- | --- | --- | --- | --- | --- | --- | --- | --- | --- | --- | --- | --- | --- | --- | --- | --- | --- | --- | --- | --- | --- | --- | --- | --- | --- | --- | --- | --- | --- | --- | --- | --- | --- | --- | --- | --- | --- | --- | --- | --- | --- | --- | --- | --- | --- | --- | --- | --- | --- | --- | --- | --- | --- | --- | --- | --- | --- | --- | --- | --- | --- | --- | --- | --- | --- | --- | --- | --- | --- | --- | --- | --- | --- | --- | --- | --- | --- | --- | --- | --- | --- | --- | --- | --- | --- | --- | --- | --- | --- | --- | --- | --- | --- | --- | --- | --- | --- | --- | --- | --- | --- | --- | --- | --- | --- | --- | --- | --- | --- | --- | --- | --- | --- | --- | --- | --- | --- | --- | --- | --- | --- | --- | --- | --- | --- | --- | --- | --- | --- | --- | --- | --- | --- | --- | --- | --- | --- | --- | --- | --- | --- | --- | --- | --- | --- | --- | --- | --- | --- | --- | --- | --- | --- | --- | --- | --- | --- | --- | --- | --- | --- | --- | --- | --- | --- | --- | --- | --- | --- | --- | --- | --- | --- | --- | --- | --- | --- | --- | --- | --- | --- | --- | --- | --- | --- | --- | --- | --- | --- | --- | --- | --- | --- | --- | --- | --- | --- | --- | --- | --- | --- | --- | --- | --- | --- | --- | --- | --- | --- | --- | --- | --- | --- | --- | --- | --- | --- | --- | --- | --- | --- | --- | --- | --- | --- | --- | --- | --- | --- | --- | --- | --- | --- | --- | --- | --- | --- | --- | --- | --- | --- | --- | --- | --- | --- | --- | --- | --- | --- | --- | --- | --- | --- | --- | --- | --- | --- | --- | --- | --- | --- | --- | --- | --- | --- | --- | --- | --- | --- | --- | --- | --- | --- | --- | --- | --- | --- | --- | --- | --- | --- | --- | --- | --- | --- | --- | --- | --- | --- | --- | --- | --- | --- | --- | --- | --- | --- | --- | --- | --- | --- | --- | --- | --- | --- | --- | --- | --- | --- | --- | --- | --- | --- | --- | --- | --- | --- | --- | --- | --- | --- | --- | --- | --- | --- | --- | --- | --- | --- | --- | --- | --- |

∞p<0.1; *p<0.05; **p<0.01; ***p<0.001

≠ Coefficient scaled to reflect change in outcome for every 1km increase in distance; ¥ Coefficient scaled to reflect change in outcome for every 100cpm increase total PA; § Coefficient scaled to reflect change in outcome for every 10% point increase in natural space

± Unadjusted bivariate association between % natural land/private gardens and SDQ outcome

NS: Natural space extraction only; NS & PG: Natural Space and Private gardens

| \| 800m Buffer \| Emotional Problems \| \| Conduct Problems \| \| Hyperactivity \| \| Peer Relationship Problems \| \| Prosocial behaviour \| \| Total SDQ score \| \| \| --- \| --- \| --- \| --- \| --- \| --- \| --- \| --- \| --- \| --- \| --- \| --- \| --- \| \|  \| NS \| NS & PG \| NS \| NS & PG \| NS \| NS & PG \| NS \| NS & PG \| NS \| NS & PG \| NS \| NS & PG \| \| **Unadjusted^±^**^§^ \| 0.00  (-0.08, 0.07) \| -0.02  (-0.13, 0.09) \| -0.03  (-0.08, 0.02) \| -0.06  (-0.14, 0.01) \| 0.03  (-0.05, 0.12) \| -0.01  (-0.15, 0.13) \| 0.02  (-0.04, 0.09) \| 0.00  (-0.12, 0.11) \| 0.03  (-0.04, 0.10) \| 0.04  (-0.07, 0.15) \| 0.02  (-0.17, 0.20) \| -0.10  (-0.42, 0.21) \| \| **Adjusted** \|  \|  \|  \|  \|  \|  \|  \|  \|  \|  \|  \|  \| \| Sweep 7 score \| 0.22***  (0.09, 0.34) \| 0.22  (0.09, 0.34) \| 0.27***  (0.16, 0.39) \| 0.27  (0.16, 0.39) \| 0.31***  (0.22, 0.41) \| 0.31  (0.22, 0.41) \| 0.24**  (0.09, 0.39) \| 0.24  (0.09, 0.39) \| 0.16*  (0.04, 0.28) \| 0.16  (0.04, 0.29) \| 0.70***  (0.62, 0.79) \| 0.70  (0.62, 0.79) \| \| Income  (ref: lowest) \|  \|  \|  \|  \|  \|  \|  \|  \|  \|  \|  \|  \| \| 2nd Quintile \| 0.26  (-0.42, 0.93) \| 0.26  (-0.42, 0.94) \| -0.13  (-0.52, 0.26) \| -0.11  (-0.50, 0.28) \| -0.46  (-1.21, 0.28) \| -0.47  (-1.20, 0.27) \| -0.24  (-0.87, 0.40) \| -0.21  (-0.85, 0.43) \| -0.08  (-0.57, 0.41) \| -0.11  (-0.60, 0.39) \| 0.33  (-0.98, 1.64) \| 0.32  (-0.97, 1.61) \| \| 3rd Quintile \| -0.02  (-0.52, 0.48) \| -0.01  (-0.52, 0.50) \| -0.12  (-0.44, 0.20) \| -0.10  (-0.42, 0.22) \| -0.34  (-0.94, 0.26) \| -0.34  (-0.94, 0.26) \| -0.35  (-0.98, 0.28) \| -0.31  (-0.95, 0.32) \| -0.01  (-0.45, 0.44) \| -0.05  (-0.49, 0.38) \| 0.09  (-1.01, 1.19) \| 0.08  (-0.98, 1.15) \| \| 4th Quintile \| -0.06  (-0.53, 0.42) \| -0.05  (-0.54, 0.44) \| -0.36*  (-0.67,-0.04) \| -0.34*  (-0.65,-0.02) \| -0.60∞  (-1.29, 0.09) \| -0.60∞  (-1.29, 0.08) \| -0.59*  (-1.18, 0.00) \| -0.55∞  (-1.15, 0.05) \| 0.17  (-0.28, 0.62) \| 0.12  (-0.32, 0.56) \| -0.15  (-1.06, 1.03) \| -0.03  (-1.04, 0.98) \| \| Highest Quintile \| -0.16  (-0.60, 0.28) \| -0.15  (-0.61, 0.30) \| -0.47*  (-0.76,-0.17) \| -0.44*  (-0.73,-0.15) \| -0.61∞  (-1.24, 0.01) \| -0.61∞  (-1.23, 0.02) \| -0.62*  (-1.18,-0.65) \| -0.58*  (-1.14,-0.01) \| 0.23  (-0.24, 0.69) \| 0.17  (-0.27, 0.62) \| -0.36  (-1.45, 0.73) \| -0.36  (-1.39, 0.67) \| \| Urban \|  \|  \|  \|  \|  \|  \|  \|  \|  \|  \|  \|  \| \| (ref:Rural) \| 0.03  (-0.57, 0.63) \| 0.01  (-0.55, 0.57) \| -0.13  (-0.53, 0.27) \| -0.09  (-0.46, 0.28) \| 0.00  (-0.64, 0.64) \| -0.10  (-0.69, 0.49) \| -0.45  (-1.04, 0.15) \| -0.49∞  (-1.03, 0.06) \| 0.45∞  (-0.02, 0.92) \| 0.34  (-0.12, 0.80) \| 0.30  (-0.81, 1.40) \| -0.01  (-0.96, 0.94) \| \| Mothers age at birth of child \| -0.02  (-0.04, 0.00) \| -0.02  (-0.04, 0.00) \| -0.01  (-0.03, 0.01) \| -0.01  (-0.03, 0.01) \| -0.03  (-0.07, 0.02) \| -0.03  (-0.07, 0.02) \| 0.00  (-0.03, 0.03) \| 0.00  (-0.03, 0.03) \| -0.01  (-0.03, 0.01) \| -0.01  (-0.03, 0.01) \| 0.06*  (0.01, 0.11) \| 0.06*  (0.01, 0.11) \| \|  \|  \|  \|  \|  \|  \|  \|  \|  \|  \|  \|  \|  \| \| Distance to School^≠^ \| 0.02  (-0.02, 0.07) \| 0.02  (-0.02, 0.07) \| -0.02∞  (-0.04, 0.00) \| -0.02*  (-0.04, 0.00) \| 0.02  (-0.04, 0.07) \| 0.02  (-0.03, 0.07) \| 0.00  (-0.03, 0.03) \| 0.00  (-0.04, 0.03) \| 0.02∞  (0.00, 0.04) \| 0.02∞  (0.00, 0.04) \| 0.02  (-0.04, 0.09) \| 0.03  (-0.03, 0.10) \| \|  \|  \|  \|  \|  \|  \|  \|  \|  \|  \|  \|  \|  \| \| Female (ref: Male) \| 0.17  (-0.21, 0.55) \| 0.17  (-0.21, 0.55) \| -0.13  (-0.37, 0.11) \| -0.13  (-0.37, 0.11) \| -0.81**  (-1.32 -0.31) \| -0.82*  (-1.33,-0.30) \| -0.13  (-0.51, 0.24) \| -0.14  (-0.51, 0.24) \| 0.60***  (0.30, 0.90) \| 0.60***  (0.29, 0.90) \| 0.07  (-0.66, 0.80) \| 0.05  (-0.69, 0.79) \| \|  \|  \|  \|  \|  \|  \|  \|  \|  \|  \|  \|  \|  \| \| Total PA^¥^ \| -0.04  (-0.12, 0.05) \| -0.04  (-0.12, 0.05) \| 0.05  (-0.02, 0.11) \| 0.05  (-0.02, 0.11) \| 0.13*  (0.01, 0.24) \| 0.13*  (0.00, 0.25) \| -0.03  (-0.12, 0.06) \| -0.03  (-0.12, 0.06) \| 0.08∞  (-0.01, 0.17) \| 0.08∞  (-0.01, 0.18) \| 0.19*  (0.02, 0.36) \| 0.19*  (0.02, 0.36) \| \|  \|  \|  \|  \|  \|  \|  \|  \|  \|  \|  \|  \|  \| \| % natural space^§^ \| 0.00  (-0.10, 0.10) \| -0.01  (-0.16, 0.13) \| -0.04  (-0.12, 0.03) \| -0.05  (-0.16, 0.05) \| 0.03  (-0.09, 0.10) \| 0.00  (-0.17, 0.18) \| -0.04  (-0.13, 0.05) \| -0.09  (-0.22, 0.04) \| 0.09∞  (0.00, 0.19) \| 0.10  (-0.03, 0.23) \| 0.10  (-0.14, 0.33) \| 0.00  (-0.30, 0.30) \| \| Constant \| 1.71  (0.34, 3.09) \| 1.80  (0.15, 3.45) \| 1.38  (0.50, 2.26) \| 1.49  (0.37, 2.60) \| 2.52  (0.33, 4.72) \| 2.75  (0.32, 5.18) \| 2.16  (0.67, 3.66) \| 2.60  (0.90, 4.32) \| 5.89  (4.12, 7.66) \| 5.74  (3.72, 7.75) \| -2.35  (-5.51, 0.81) \| -1.63  (-5.25, 2.00) \| \| Observations \| 726 \| 726 \| 726 \| 726 \| 725 \| 725 \| 724 \| 724 \| 726 \| 726 \| 725 \| 725 \| \| Population size \| 731 \| 731 \| 731 \| 731 \| 730 \| 30 \| 728 \| 728 \| 731 \| 731 \| 726 \| 726 \| \| F Statistic \| 2.06* \| 2.06* \| 4.66*** \| 4.69*** \| 9.45*** \| 5.16*** \| 2.16* \| 2.58* \| 2.93** \| 2.55* \| 27.28*** \| 26.94*** \| \| Population R^2^ \| 0.06 \| 0.06 \| 0.12 \| 0.12 \| 0.17 \| 0.08 \| 0.07 \| 0.08 \| 0.11 \| 0.10 \| 0.52 \| 0.52 \| |
| --- | --- | --- | --- | --- | --- | --- | --- | --- | --- | --- | --- | --- | --- | --- | --- | --- | --- | --- | --- | --- | --- | --- | --- | --- | --- | --- | --- | --- | --- | --- | --- | --- | --- | --- | --- | --- | --- | --- | --- | --- | --- | --- | --- | --- | --- | --- | --- | --- | --- | --- | --- | --- | --- | --- | --- | --- | --- | --- | --- | --- | --- | --- | --- | --- | --- | --- | --- | --- | --- | --- | --- | --- | --- | --- | --- | --- | --- | --- | --- | --- | --- | --- | --- | --- | --- | --- | --- | --- | --- | --- | --- | --- | --- | --- | --- | --- | --- | --- | --- | --- | --- | --- | --- | --- | --- | --- | --- | --- | --- | --- | --- | --- | --- | --- | --- | --- | --- | --- | --- | --- | --- | --- | --- | --- | --- | --- | --- | --- | --- | --- | --- | --- | --- | --- | --- | --- | --- | --- | --- | --- | --- | --- | --- | --- | --- | --- | --- | --- | --- | --- | --- | --- | --- | --- | --- | --- | --- | --- | --- | --- | --- | --- | --- | --- | --- | --- | --- | --- | --- | --- | --- | --- | --- | --- | --- | --- | --- | --- | --- | --- | --- | --- | --- | --- | --- | --- | --- | --- | --- | --- | --- | --- | --- | --- | --- | --- | --- | --- | --- | --- | --- | --- | --- | --- | --- | --- | --- | --- | --- | --- | --- | --- | --- | --- | --- | --- | --- | --- | --- | --- | --- | --- | --- | --- | --- | --- | --- | --- | --- | --- | --- | --- | --- | --- | --- | --- | --- | --- | --- | --- | --- | --- | --- | --- | --- | --- | --- | --- | --- | --- | --- | --- | --- | --- | --- | --- | --- | --- | --- | --- | --- | --- | --- | --- | --- | --- | --- | --- | --- | --- | --- | --- | --- | --- | --- | --- | --- | --- | --- | --- | --- | --- | --- | --- | --- | --- | --- | --- | --- | --- | --- | --- | --- | --- | --- | --- | --- | --- | --- | --- | --- | --- | --- | --- | --- | --- | --- | --- | --- | --- | --- | --- | --- | --- | --- | --- | --- | --- | --- | --- | --- | --- | --- | --- | --- | --- | --- | --- | --- | --- | --- | --- | --- | --- | --- | --- | --- | --- |

∞p<0.1; *p<0.05; **p<0.01; ***p<0.001

≠ Coefficient scaled to reflect change in outcome for every 1km increase in distance; ¥ Coefficient scaled to reflect change in outcome for every 100cpm increase total PA; § Coefficient scaled to reflect change in outcome for every 10% point increase in natural space

± Unadjusted bivariate association between % natural land/private gardens and SDQ outcome

NS: Natural space extraction only; NS & PG: Natural Space and Private gardens

| **100m and 800 Buffer - PG** | Emotional Problems | | Conduct Problems | | Hyperactivity | | Peer Relationship Problems | | Prosocial behaviour | | Total SDQ score | |
| --- | --- | --- | --- | --- | --- | --- | --- | --- | --- | --- | --- | --- |
|  | 100m | 800m | 100m | 800m | 100m | 800m | 100m | 800m | 100m | 800m | 100m | 800m |
| **Unadjusted±**§ | -0.02  (-0.14, 0.09) | -0.03  (-0.18, 0.12) | 0.00  (-0.09, 0.08) | 0.01  (-0.08, 0.11) | -0.10  (-0.27, 0.07) | -0.17  (-0.34, 0.01) | -0.05  (-0.17, 0.08) | -0.12  (-0.25, 0.01) | -0.08  (-0.18, 0.02) | -0.05  (-0.18, 0.09) | -0.20  (-0.58, 0.18) | -0.30  (-0.64, 0.03) |
| **Adjusted** |  |  |  |  |  |  |  |  |  |  |  |  |
| Sweep 7 score | 0.21***  (0.09, 0.34) | 0.22***  (0.09, 0.34) | 0.27***  (0.16, 0.38) | 0.27***  (0.16, 0.38) | 0.31***  (0.22, 0.41) | 0.31***  (0.22, 0.41) | 0.23**  (0.08, 0.38) | 0.23**  (0.08, 0.38) | 0.15*  (0.02, 0.28) | 0.16***  (0.03, 0.28) | 0.70***  (0.62, 0.79) | 0.70***  (0.62, 0.79) |
| Income  (ref: bottom) |  |  |  |  |  |  |  |  |  |  |  |  |
| 2nd Quintile | 0.25  (-0.43, 0.94) | 0.26  (-0.42, 0.95) | -0.13  (-0.53, 0.27) | -0.14  (-0.53, 0.25) | -0.45  (-1.19, 0.28) | -0.43  (-1.18, 0.31) | -0.23  (-0.87, 0.42) | -0.23  (-0.87, 0.42) | -0.06  (-0.57, 0.44) | -0.04  (-0.55, 0.46) | 0.35  (-0.97, 1.66) | 0.43  (-0.85, 1.70) |
| 3rd Quintile | -0.02  (-0.54, 0.49) | -0.01  (-0.51, 0.50) | -0.13  (-0.45, 0.20) | -0.15  (-0.48, 0.19) | -0.31  (-0.91, 0.29) | -0.28  (-0.89, 0.33) | -0.34  (-0.97, 0.29) | -0.34  (-0.96, 0.29) | 0.03  (-0.41, 0.47) | 0.06  (-0.42, 0.54) | 0.13  (-1.01, 1.28) | 0.26  (-0.82, 1.34) |
| 4th Quintile | -0.06  (-0.55, 0.42) | -0.05  (-0.52, 0.43) | -0.36*  (-0.68, 0.04) | -0.38*  (-0.71, 0.05) | -0.58  (-1.27, 0.11) | -0.55  (-1.24, 0.14) | -0.58  (-1.17, 0.01) | -0.57  (-1.16, 0.01) | 0.20  (-0.26, 0.66) | 0.23  (-0.26, 0.72) | 0.02  (-1.05, 1.09) | 0.15  (-0.87, 1.16) |
| Top Quintile | -0.17  (-0.62, 0.29) | -0.15  (-0.59, 0.29) | -0.47**  (-0.79, 0.16) | -0.49**  (-0.80, 0.18) | -0.58  (-1.20, 0.05) | -0.55  (-1.18, 0.07) | -0.61*  (-1.18, -0.04) | -0.61*  (-1.16, -0.06) | 0.28  (-0.21, 0.77) | 0.30  (-0.22, 0.82) | -0.30  (-1.45, 0.86) | -0.17  (-1.24, 0.91) |
| Urban |  |  |  |  |  |  |  |  |  |  |  |  |
| (ref:Rural) | 0.02  (-0.48, 0.51) | 0.06  (-0.48, 0.60) | -0.01  (-0.30, 0.51) | -0.07  (-0.41, 0.28) | -0.05  (-0.49, 0.40) | -0.05  (-0.51, 0.62) | -0.29  (-0.80, 0.22) | -0.27  (-0.84, 0.30) | 0.23  (-0.12, 0.57) | 0.34  (-0.04, 0.72) | 0.07  (-0.69, 0.83) | 0.47  (-0.52, 1.46) |
| Mothers age at birth of child | -0.02  (-0.04, 0.00) | -0.02  (-0.04, 0.00) | -0.01  (-0.03, 0.01) | -0.01  (-0.03, 0.01) | -0.03  (-0.07, 0.02) | -0.03  (-0.07, 0.01) | 0.00  (-0.03, 0.02) | 0.00  (-0.03, 0.02) | 0.00  (-0.02, 0.02) | -0.01  (-0.03, 0.01) | 0.06*  (0.01, 0.11) | 0.06*  (0.01, 0.11) |
|  |  |  |  |  |  |  |  |  |  |  |  |  |
| Distance to School≠ | 0.03  (-0.02, 0.07) | 0.02  (-0.02, 0.06) | -0.02  (-0.05, 0.00) | -0.02  (-0.04, 0.00) | 0.02  (-0.03, 0.06) | 0.02  (-0.04, 0.07) | -0.01  (-0.04, 0.02) | -0.01  (-0.04, 0.02) | 0.02  (0.00, 0.05) | 0.02  (0.00, 0.05) | 0.02  (-0.04, 0.09) | 0.02  (-0.04, 0.08) |
|  |  |  |  |  |  |  |  |  |  |  |  |  |
| Female (ref: Male) | 0.17  (-0.20, 0.55) | 0.17  (-0.22, 0.56) | -0.12  (-0.36, 0.12) | -0.13  (-0.36, 0.11) | -0.83**  (-1.34, -0.32) | -0.82**  (-1.33, -0.31) | -0.13  (-0.50, 0.24) | -0.12  (-0.50, 0.25) | 0.57***  (0.29, 0.86) | 0.58***  (0.29, 0.88) | 0.04  (-0.70, 0.78) | 0.06  (-0.67, 0.80) |
|  |  |  |  |  |  |  |  |  |  |  |  |  |
| Total PA^¥^ | -0.03  (-0.12, 0.05) | -0.04  (-0.12, 0.05) | 0.05  (-0.02, 0.11) | 0.05  (-0.02, 0.11) | 0.12*  (0.00, 0.24) | 0.13*  (0.01, 0.25) | -0.03  (-0.13, 0.06) | -0.03  (-0.12, 0.06) | 0.08  (-0.01, 0.17) | 0.08  (-0.01, 0.17) | 0.19*  (0.01, 0.36) | 0.19*  (0.02, 0.36) |
|  |  |  |  |  |  |  |  |  |  |  |  |  |
| % natural space^§^ | 0.02  (-0.11, 0.15) | -0.02  (-0.19, 0.15) | 0.02  (-0.08, 0.11) | 0.06  (-0.08, 0.19) | -0.06  (-0.23, 0.11) | -0.12  (-0.36, 0.13) | -0.02  (-0.16, 0.12) | -0.03  (-0.21, 0.15) | -0.10∞  (-0.20, 0.00) | -0.15∞  (-0.33, 0.03) | -0.10  (-0.36, 0.16) | -0.36∞  (-0.75, 0.03) |
| Constant | 1.66  (0.42, 2.89) | 1.71  (0.54, 2.89) | 1.01  (0.21, 1.81) | 1.01  (0.28, 1.73) | 2.91  (0.98, 4.85) | 2.86  (1.12, 4.61) | 1.90  (0.27, 3.52) | 1.87  (0.35, 3.39) | 6.91  (5.29, 8.54) | 6.78  (5.12, 8.44) | -1.36  (-3.62, 0.90) | -1.33  (-3.34, 0.68) |
| Observations | 726 | 726 | 726 | 726 | 725 | 725 | 724 | 724 | 726 | 726 | 725 | 725 |
| Population size | 731 | 731 | 731 | 731 | 730 | 730 | 728 | 728 | 731 | 731 | 726 | 726 |
| F Statistic | 2.27 | 2.14 | 4.72 | 4.68 | 9.58 | 9.76 | 2.33 | 2.31 | 2.86 | 3.40 | 33.9 | 28.5 |
| Population R^2^ | 0.06 | 0.06 | 0.12 | 0.12 | 0.17 | 0.17 | 0.07 | 0.07 | 0.10 | 0.10 | 0.52 | 0.53 |

∞p<0.1; *p<0.05; **p<0.01; ***p<0.001

≠ Coefficient scaled to reflect change in outcome for every 1km increase in distance; ¥ Coefficient scaled to reflect change in outcome for every 100cpm increase total PA; § Coefficient scaled to reflect change in outcome for every 10% point increase in natural space

± Unadjusted bivariate association between % natural land/private gardens and SDQ outcome

NS: Natural space extraction only; NS & PG: Natural Space and Private gardens


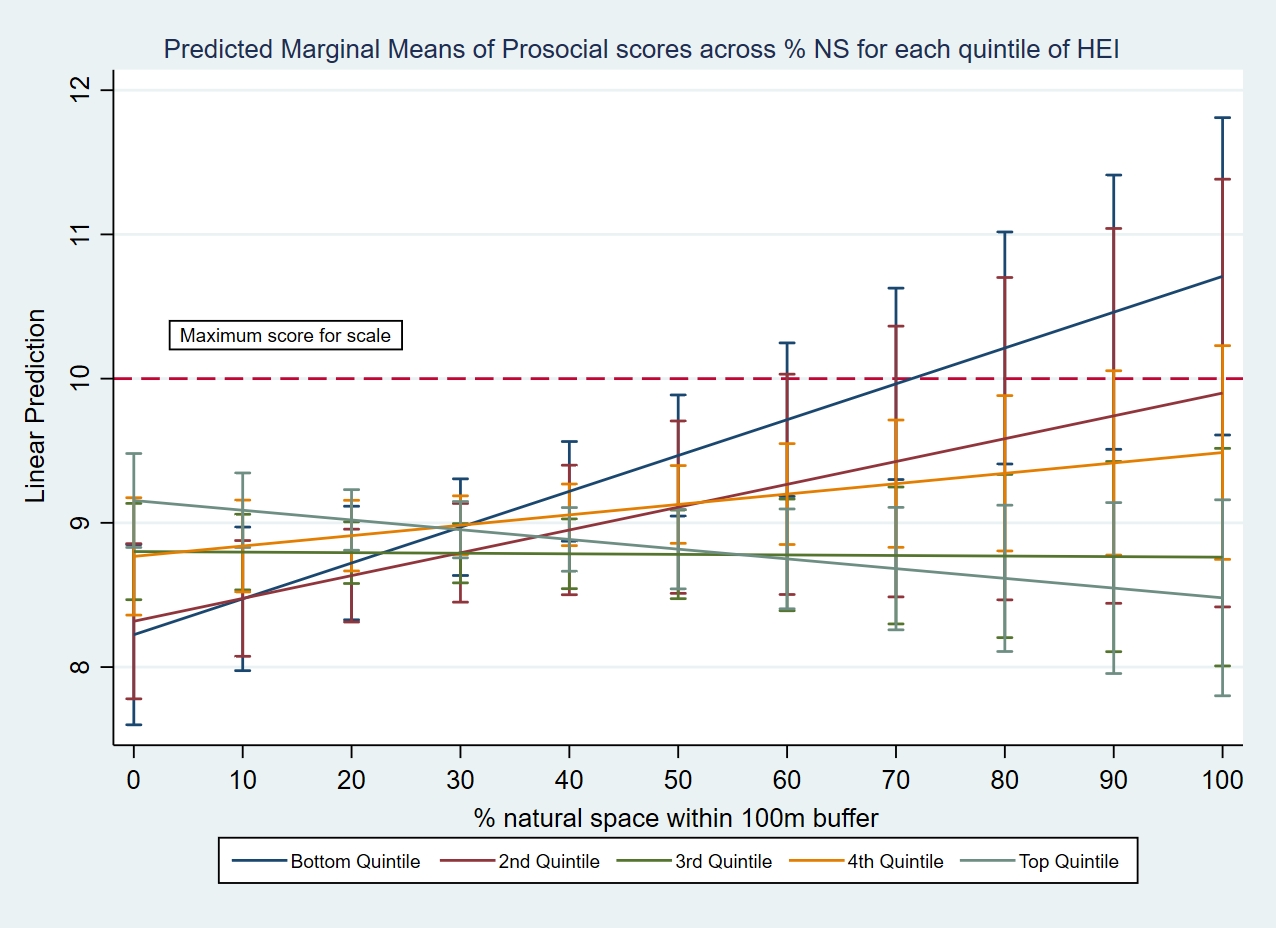

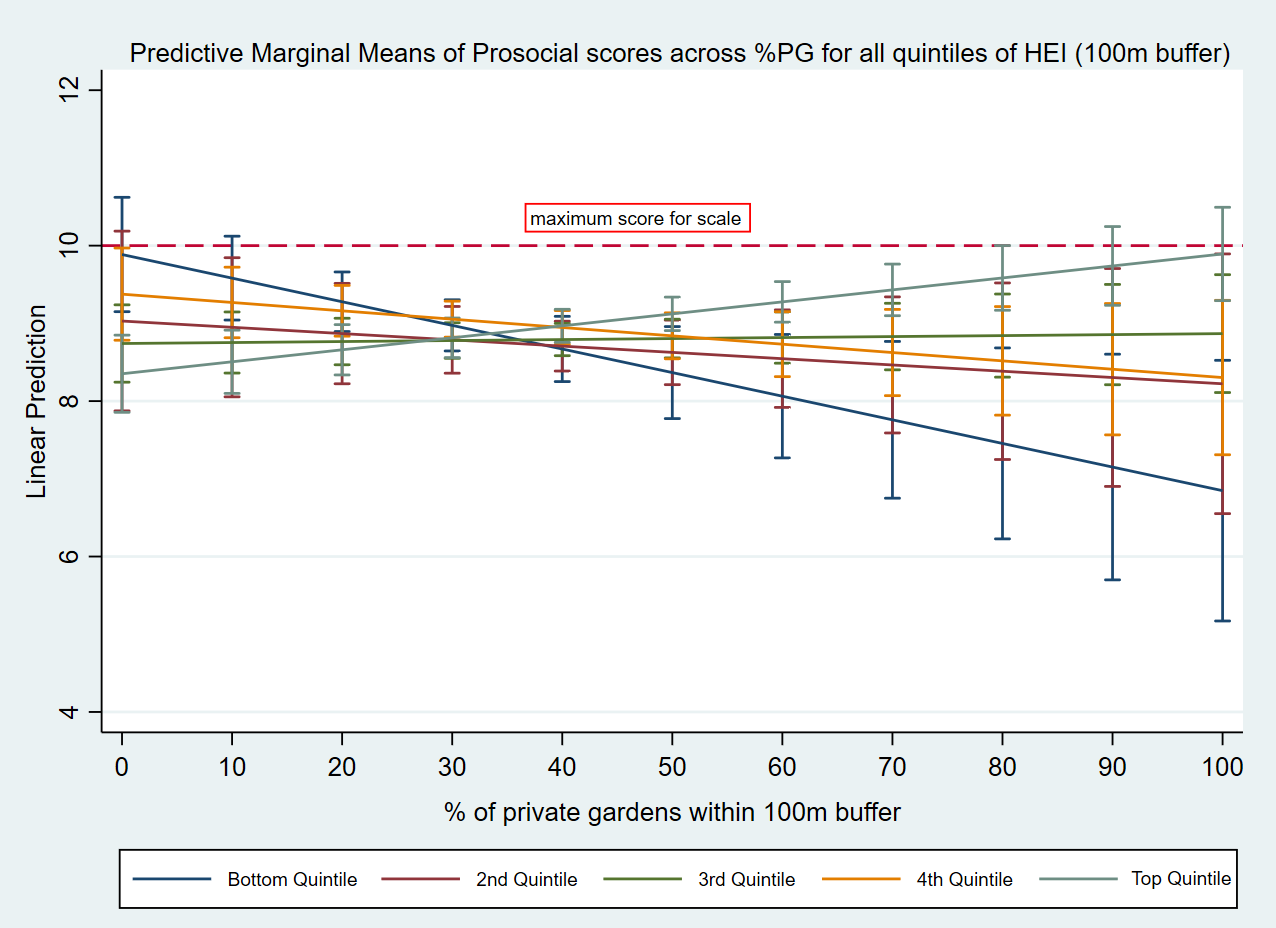

Supplement: Supplementary file 2 [file mmc2.docx]
